# A proof‐of‐concept experimental study for vacuum‐driven anaerobic biosolids fermentation using the IntensiCarb technology

**DOI:** 10.1002/wer.10694

**Published:** 2022-03-03

**Authors:** Frances Okoye, Farokh Laqa Kakar, Elsayed Elbeshbishy, Kati Bell, Christopher Muller, Jose Jimenez, Ahmed Al‐Omari, Domenico Santoro, Eunkyung Jang, John Walton, Gholamreza Bahreini, Masuduz Zaman, George Nakhla, Ferenc Hazi, Imre Takacs, Sudhir Murthy, Diego Rosso

**Affiliations:** ^1^ Environmental Research for Resource Recovery Group, Department of Civil Engineering Ryerson University Toronto Canada; ^2^ Brown and Caldwell Walnut Creek California USA; ^3^ USP Technologies London Canada; ^4^ Department of Civil and Environmental Engineering Western University London Canada; ^5^ Dynamita Sigale France; ^6^ NEWHub Corp Herndon Virginia USA; ^7^ Civil and Environmental Engineering Department University of California Irvine California USA; ^8^ Water‐Energy Nexus (WEX) Center University of California Irvine California USA

**Keywords:** fermentation, intensification, resource recovery, sludge treatment, thickening, vacuum evaporation

## Abstract

This study demonstrates the potential of an innovative anaerobic treatment technology for municipal biosolids (IntensiCarb), which relies on vacuum evaporation to decouple solids and hydraulic retention times (SRT and HRT). We present proof‐of‐concept experiments using primary sludge and thickened waste activated sludge (50–50 v/v mixture) as feed for fermentation and carbon upgrading with the IntensiCarb unit. IntensiCarb fully decoupled the HRT and SRT in continuously stirred anaerobic reactors (CSAR) to achieve two intensification factors, that is, 1.3 and 2, while keeping the SRT constant at 3 days (including in the control fermenter). The intensified CSARs were compared to a conventional control system to determine the yields of particulate hydrolysis, VFA production, and nitrogen partitioning between fermentate and condensate. The intensified CSAR operating at an intensification factor 2 achieved a 65% improvement in particulate solubilization. Almost 50% of total ammonia was extracted without pH adjustment, while carbon was retained in the fermentate. Based on these results, the IntensiCarb technology allows water resource recovery facilities to achieve a high degree of plant‐wide intensification while partitioning nutrients into different streams and thickening solids.

**Practitioner Points:**

- The IntensiCarb reactor can decouple hydraulic (HRT) and solids (SRT) retention times in anaerobic systems while also increasing particulate hydrolysis and overall plant capacity.
- Using vacuum as driving force of the IntensiCarb technology, the system could achieve thickening, digestion, and partial dewatering in the same unit—thus eliminating the complexity of multi‐stage biosolids treatment lines.
- The ability to partition nutrients between particulate, fermentate, and condensate assigns to the IntensiCarb unit a key role in recovery strategies for value‐added products such as nitrogen, phosphorus, and carbon, which can be recovered separately and independently.

## Graphical abstract

Intermittent feed and vacuum extraction with the IntensiCarb reactor provide an avenue to recover water and volatile organics, while minimizing reactor volume.

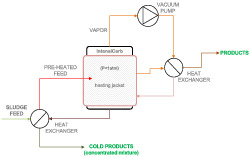

## INTRODUCTION

Anaerobic sludge digestion technologies have been successfully applied to municipal waste since the 19th century to stabilize organic waste, odor control, volume reduction, and biogas generation (Lusk et al., 1996). Anaerobic digestion (AD) comprises multiple microbially driven interdependent stages that are sensitive to environmental and operational parameters, including particulate hydrolysis, fermentation, and methanogenesis (Appels et al., 2008). Due to low biomass growth rates, long retention times are essential for adequate microbial growth and organics stabilization during AD. This has led to large spatial requirements and capital expenditures within the water resource recovery facilities (WRRFs) that decide to apply this technology (McLeod et al., 2019). These considerations have limited the adoption potential of AD to only large utilities (treating >5 million gallons of wastewater per day), with more than 30% of these utilities flaring the biogas in the absence of meaningful use for it (Tanigwa, 2017). The custom of wasting biogas displays poor management strategies of carbon resources in current WWRFs, which are critical to overcoming cost and sustainability issues for advanced nutrient removal.

Partial AD, also known as sludge fermentation, involves converting particulate substrate to soluble organic compounds (hydrolysis) and further conversion to volatile fatty acids (VFAs), hydrogen, and carbon dioxide. Fermentation will occur at shorter retention times, under 5 days; methanogenesis would inevitably take place at longer times (Appels et al., 2008). Although not widely implemented in the field, fermentation can be critical in improving carbon management strategies at a plant‐wide scale. Specifically, VFAs can be efficiently generated with fermentation and diverted for use as an internal carbon source for biological nutrient removal (BNR), to produce biopolymers (polyhydroxyalkanoates), and for bio‐electrochemical treatment processes (Raheem et al., 2018; Yesil et al., 2014). Indeed, BNR processes are typically supplemented with methanol, ethanol, acetate, or glucose when the carbon in the influent wastewater is insufficient, increasing wastewater treatment costs considerably (Zhang et al., 2015).

Conventional anaerobic processes are typically low‐rate processes. Intensification strategies to manage this limitation have been pursued primarily by increasing the concentration of solids in the digesters through feed sludge thickening by centrifuges, gravity belt thickeners, thickening tanks, polymer use, and so forth, before AD (Jin & Parker, 2017). Solids concentrations can also be increased by decoupling the solids retention time (SRT) from the hydraulic retention time (HRT) in the digesters using recuperative thickening (RT), a strategy by which thickened sludge is partially returned to the digester (Li et al., 2020). This technique has improved AD capacity by more than 200%, enhanced biogas production by 20%, and volatile solids reduction (VSR) by 22% in a full‐scale application (Bharambe et al., 2015). However, RT adds mechanical complexity and increased expense for the WRRF. Intensification can also be achieved by sludge pretreatment to improve the hydrolysis stage of AD. Pretreatment increases the bioavailability of organic matter already present in the sludge. Sludge pretreatment can use thermal, biological, mechanical, and chemical methods (Carrère et al., 2010). Of these techniques, thermal hydrolysis is well researched and has been adopted commercially for over 20 years. The technique requires high pressures and elevated temperatures (160–180°C) to pretreat the feed sludge. When applied before fermentation, thermal hydrolysis resulted in up to two times the increase in VFA yield with SRT reduction from 5.8 to 2.2 days with no negative impact (Morgan‐Sagastume et al., 2011). While thermal hydrolysis can increase digester capacity, the technique has significant energy requirements and the potential to produce refractory by‐products (Carrère et al., 2010).

The IntensiCarb™ (IC) system, a patent‐pending technology co‐developed by USP Technologies and Brown and Caldwell, approaches process intensification through an efficient solid–liquid separation process enabled by vacuum evaporation at temperatures (20–60°C) that are compatible with anaerobic processes. This technology integrates thickening, digestion, and partial dewatering into a single unit where biochemical and physical treatment rates are simultaneously optimized, including (a) the rate of biochemical activities among and within the liquid, the solid, and the gaseous fractions of the biosolids subjected to treatment; (b) the rate of mass transfer among and within the liquid, the solid, and the gaseous fractions; and (c) the rate of retention of the particulate and non‐volatile fractions of the biosolids in the treatment system.

Moreover, the IntensiCarb system leverages the mature technology of industrial vacuum evaporators. However, while industrial vacuum evaporators are established on accelerating the time required for water evaporation, in the IntensiCarb technology, biochemical reactions are promoted and driven by vacuum pressures in combination with the typical physicochemical processes induced by evaporation. Under vacuum conditions, water can be evaporated from biosolids at temperatures compatible with biochemical reactions (20–60°C), promoting intensification. The IntensiCarb technology also integrates ancillary units for heat recovery, which allows operation with a power demand in the range of 25–75 kWh per wet ton of treated biosolids. Selective extraction of dissolved constituents and water will enable solids to be retained entirely in the IntensiCarb reactor for an extended and arbitrary period, effectively decoupling the HRT and SRT without negative impact on the anaerobes while enhancing hydrolysis of particulate organic matter (Rajhi et al., 2016). Volatile compounds such as VFAs and ammonia can also be selectively driven out from the reactor and collected in the condensate by controlling the pH. For example, up to 93% of ammonia was stripped when the pH of the reactor was above 9 (Bonmatí & Flotats, 2003; Tao et al., 2018).

The IntensiCarb operates according to the following elementary treatment steps:

- Extraction and transport of the biosolids from the clarifiers to the IntensiCarb unit.
- Vacuum‐driven thickening of the biosolids in the IntensiCarb unit.
- Simultaneous fermentation and digestion of biosolids, assisted by vacuum.
- Continuous dewatering of biosolids, assisted by vacuum.
- pH‐controlled nutrient partitioning and recovery in the condensate.

In this study, a laboratory‐scale proof‐of‐concept of the IntensiCarb technology was achieved. New knowledge was developed, and our understanding of the operational conditions of the IntensiCarb technology expanded to intensification factors up to 2 (i.e., 100% reduction in hydraulic retention time). The composition of the fermentate and condensate was evaluated, specifically in terms of ammonia, sCOD, and VFA content. The critical process yields associated with the control (non‐intensified, conventional fermented) and the IntensiCarb intensified scenarios were compared and discussed.

## MATERIALS AND METHODS

### Sludge source and composition

The wastewater sludge used for this study was obtained from Ashbridges Bay Wastewater Treatment Plant, the second largest WRRF in Canada located in Toronto, Ontario. Primary sludge and thickened waste activated sludge were collected from the facility weekly. Both types of sludge were combined in a 50:50 v/v ratio to create mixed sludge (MS), the feedstock into the laboratory fermenter. MS was stored in a refrigerator at 4°C in polyethylene containers until use. Digestate from anaerobic digester #7 was used to inoculate the fermenters at the same facility. The digesters at the facility were operated at 20‐day SRT under mesophilic conditions. Before inoculation, the digestate was heated to 70°C for 30 min to inactivate the methanogens. Characteristics of the different sludge are shown in Table 1.

**TABLE 1:** Characteristics of feedstock and digestate

Parameters	Mixed sludge (*n* = 9^a^)	Digestate (*n* = 3^a^)
TCOD (mg/l)	36,900 ± 4,200	23,000 ± 2,400
sCOD (mg/l)	1,540 ± 690	2,920 ± 40
TN (mg/l)	1,650 ± 360	1,450 ± 70
sN (mg/l)	120 ± 30	640 ± 10
NH_4_‐N (mg/l)	82 ± 15	480 ± 10
VFAs (mg COD/l)	620 ± 320	140 ± 10
TS (mg/l)	30,700 ± 3,900	21,700 ± 200
VS (mg/l)	20,400 ± 2,700	13,000 ± 300
TSS (mg/l)	27,600 ± 4,000	19,400 ± 140
VSS (mg/l)	20,500 ± 2,600	11,800 ± 140

^a^ Number of samples used to calculate averages and standard deviations.

### Wet chemistry analyses

The feedstock, fermentate, and condensate samples were monitored at least three times weekly for total, soluble, and particulate chemical oxygen demand (TCOD, sCOD, and PCOD); total and soluble nitrogen (TN and sN); ammonia‐nitrogen (NH_4_‐N); and total VFA. These parameters were analyzed using HACH Test 'N Tube methods compatible with the HACH Spectrophotometer DR/3900. Soluble parameters were obtained by centrifuging complete samples for 30 min at 9,000 RPM. The obtained centrate was then filtered using 0.45 μm syringe filters. The total volatile and suspended solids (TS, VS, TSS, and VSS) measurements were conducted using APHA standard methods 2520 B, D, E (Eaton et al., 1997).

### IntensiCarb laboratory system and equipment set‐up

**FIGURE 1:**
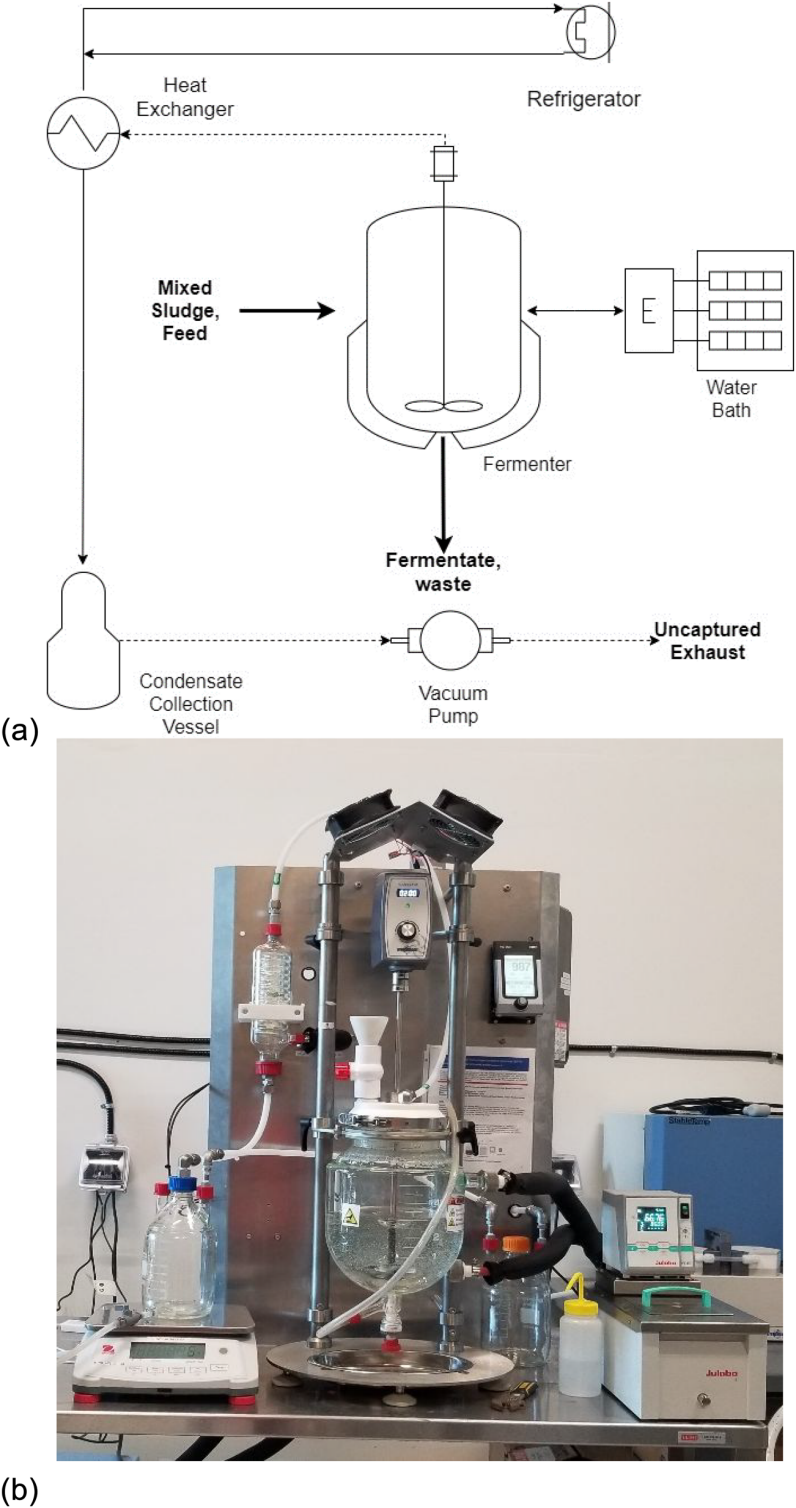
Bench‐scale IntensiCarb unit: (a) schematic diagram of the system; (b) picture of reactor set‐up

The bench‐scale IntensiCarb system comprised a 5 L double‐walled reactor which allowed hot water from the water bath to flow on one side of the wall around the reactor for temperature control. The reactor cap was fitted with a thermometer to monitor the internal temperature of the vessel, a mixing shaft that was driven by an industrial‐grade motor (Orientalmotor GFV series) with a gear ratio of 5:1 to maintain thoroughly mixed conditions within the reactor and the vacuum line to apply the desired vacuum pressure. The vacuum line was connected to the heat exchanger, which was in contact with cold water at 4°C from the refrigerator. The cold water circulating in the reactor jacket facilitated evaporate condensation from the vacuum line into a 2 L collection bottle connected after the reactor and before the vacuum pump. The system had a vacuum control unit to adjust the internal pressure in the reactor. The flow of liquid, gas, and sludge in the IntensiCarb system is shown in Figure 1.

### Operational procedure for the laboratory scale IntensiCarb unit

Semi‐continuous experiments were performed using the equipment set‐up described in the previous section, all operating at 3‐day SRT but with different HRTs of 1.5, 2.25, and 3 days. These experiments were run at an internal temperature of 60°C. The 3‐day HRT test did not require vacuum application since there was no HRT/SRT decoupling (the control representing a conventional fermenter). During the vacuum period for the other two tests, the internal pressure was maintained at 150 mbar. Evaporation heat was provided by circulating hot water in the jacketed glass vessel mimicking the IntensiCarb unit.

At the start of each experiment, the reactor was inoculated with 2 L pretreated digestate and 1 L of MS. In the 3‐day HRT test with no decoupling (i.e., the conventional fermenter), 1 L of fermentate was removed daily and replaced with 1 L of MS. For the other two experiments using vacuum as driving force, the system was run on a vacuum → waste → feed → ferment cycle. The pH of the feedstock and reactors was not adjusted to monitor the effect of the IC system without chemical addition. The vacuum was run for enough time to extract 1,500 ml (vacuum time: 7 h) and 500 ml condensate (vacuum time: 1.5 h) for the 1.5 and 2.25‐day HRT tests, respectively, corresponding to intensification factors (IF) of 2 and 1.33, respectively. Following the vacuum period, 500 and 830 ml of fermentate were wasted from the reactor corresponding to the 1.5 and 2.25‐day HRT tests. The required volume of MS to maintain 3 L of sludge within the reactor was added. The system was then fermented at 55–60°C until the following day. The duration of the experimental runs was 23, 19, and 26 days for the 1.5‐, 2.25‐, and 3‐day HRT tests, respectively. These days were over three SRTs (set at 3 days for all systems), a condition required to achieve quasi‐steady state.

### Data analysis

The IF for the three experimental runs was determined as shown in Equation 1. As a result, 1.5‐, 2.25‐, and 3‐day HRT experiments had IF of 2, 1.3, and 1, respectively. From this equation, it is shown that the IFs increased as the HRTs reduced. The data that were collected from the experiments were analyzed against these IFs.

(1) $$\text{IF}=\frac{\mathrm{SRT}}{\mathrm{HRT}}.$$

The extent to which the concentration of TS was affected by the IC unit was computed using Equation 2, where $\left\{TS_{\text{out}}\right\}$ and $\left\{TS_{\text{in}}\right\}$ refer to the average concentrations of total solids in the fermentate and MS, respectively, during the steady‐state of the experimental run. Additionally, the destruction of TSS and VSS was determined using Equation 3, where $SS_{\text{in}}$ refers to the corresponding mass of suspended solids in the MS that fed daily into the system and $SS_{\text{out}}$ is the mass of suspended solids wasted from the system daily after a steady‐state had been reached.

(2) $$\text{Thickening}\;\left(\%\right)=\frac{\left\{TS_{\text{out}}\right\}-\left\{TS_{\text{in}}\right\}}{\left\{TS_{\text{out}}\right\}}\times 100,$$

(3) $$\mathrm{SSR}\%=\frac{\left\{SS_{\text{in}}\right\}-\left\{SS_{\text{out}}\right\}}{\left\{SS_{\text{in}}\right\}}\times 100.$$

The yield of soluble organic matter and the VFAs achieved during fermentation were determined using Equations 4 and 5 (at steady‐state reactor conditions), respectively. In these equations, *PCOD*, VFA, *VSS*, and *sCOD* refer to the average daily mass of these parameters. The subscripts in and out refer to MS and the sum of fermentate and condensate, respectively.

(4) $$\text{sCOD yield}=\frac{\left\{\mathrm{PCO}D_{\text{in}}\right\}-\left\{\mathrm{PCO}D_{\text{out}}\right\}}{\left\{\mathrm{VS}S_{\text{in}}\right\}},$$

(5) $$\mathrm{VFA}\;\text{yield}=\frac{\left\{\mathrm{VF}A_{\text{out}}\right\}-\left\{\mathrm{VF}A_{\text{in}}\right\}}{\left\{\mathrm{VS}S_{\text{in}}\right\}}.$$

### Specific denitrification rate test

The potential use of the fermentate as an internal carbon source and suitable substrate was demonstrated using the specific denitrification rate (SDNR) test. The test protocol adopted was similar to that outlined by Kampas et al. (2009). Fermentates from each experiment were centrifuged at 5,000 rpm for 20 min, decanted, and the centrate was used as the substrate. The SDNR test was set up in six batch reactors with an active volume of 1.5 L. The reactors were inoculated with WAS, appropriately decanted to achieve 3 to 3.5 g/L VSS. The substrates and nitrate stock solution, prepared using sodium nitrate crystals and deionized water, were added to obtain an initial sCOD:N ratio of 7. The test was run for 4 h. Ten milliliters of sample was collected every 15 min in the first hour, every 30 min the second hour, and every hour thereafter. Samples were filtered with 0.45 μm sterile syringe filters, then nitrate–nitrogen, nitrite–nitrogen, and sCOD tests were conducted. The pH, TSS, and VSS concentrations were monitored during the experiment.

Equations 6 and 7 were used to determine the concentration of NO_x_—N and maximum SDNR, respectively. Since nitrite accumulation is significant during the test, the resulting carbon utilization should be accounted for in NO_x_ calculations. $N_{\mathrm{NOx}},N_{NO_{3}},N_{NO_{2}}$ are the concentrations of NO_x_, nitrate, and nitrite as nitrogen, respectively. VSS is the concentration of volatile suspended solids in the batch reactors at the beginning of the specific denitrification test. The *in* and *t* subscripts refer to the beginning of the experiment and the time when the most rapid stage of NO_x_ depletion concluded, respectively.

(6) $$N_{\mathrm{NOx}}=\left\{N_{NO_{3}}\right\}+\left\{N_{NO_{2}}\right\},$$

(7) $$\text{SDNR}=\frac{\left\{N_{\mathrm{NOx},\text{in}}\right\}-\left\{N_{\mathrm{NOx},t}\right\}}{\left\{\mathrm{VS}S_{\text{in}}\right\}\times t}.$$

## RESULTS AND DISCUSSIONS

### Impact of intensification factors on organics solubilization

**FIGURE 2:**
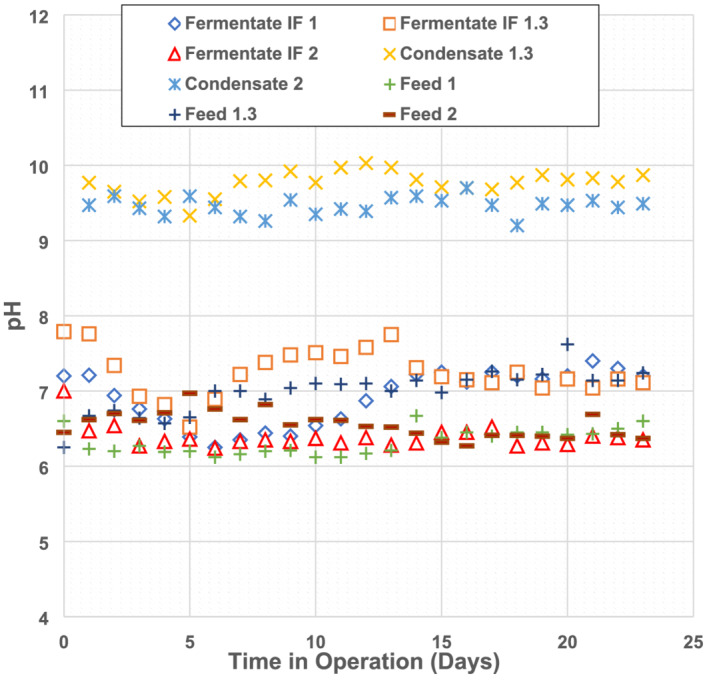
pH of feed, fermentate, and condensate from bench‐scale fermentation where IF 1 is 3‐day HRT, IF 1.3 is 2.25‐day HRT, and IF 2 is 1.5‐day HRT

Bench‐scale laboratory experiments were used to investigate the impact of intensifying fermentation using vacuum evaporation in the IntensiCarb (IC) technology. The daily measured pH over the experimental period is shown in Figure 2. The pH of MS was between 6.5 and 7.0. The control fermentate and the IF 1.3 fermentate pH were 6.2 to 7.4 and 6.5 to 7.8, respectively. The pH in the IF 2 fermentate was more acidic than the other two experiments, varying between 6.2 and 6.5. The lower pH observed could be due to higher concentrations of VFA in the fermenter during IF 2. At the relatively short SRT used, the pH variation across the fermenters is unlikely to be responsible for significant differences in organics solubilization and nutrient release since studies have shown that no changes between pH 6 and 7 during fermentation under 4‐day SRT (Chen et al., 2007). The condensate pH extracted from IF 2 and 1.3 experiments ranged from 9.2 to 9.7 and 9.3 to 10.0, showing significant overlap. The mass of various monitored parameters of feed, fermentate, and condensate for the three tests are provided in Table 2. The average daily mass balances for the total COD and nitrogen are also shown in Table 2, computed as the ratio of the sum of total COD or nitrogen in the fermentate waste and condensate collected to the total COD or nitrogen in the feed MS.

**TABLE 2:** Daily mass flow of parameters in the influent (mixed sludge) and effluents (fermentate and condensate)

	(mg/d)	TCOD	sCOD	NH_3_‐N	VFA‐COD	TS	VS	TSS	VSS
IF 1	MS	41,200	1,900	90	650	32,700	19,700	29,600	22,600
	Std^a^ (4)^b^	2,200	700	10	90	3,800	2,200	5,000	1,500
	Fermentate	36,500	8,800	830	2,300	27,200	23,120	23,600	17,400
	Std (5)	5,600	1,200	50	200	2,200	1,700	3,600	1,700
	Mass balance^c^	93%		96%					
IF 1.3	MS	42,800	1,200	110	450	38,500	25,400	29,300	25,400
	Std (4)	1,800	300	30	60	3,100	1,200	4,900	1,200
	Fermentate	38,300	9,300	470	2,400	31,100	18,300	22,200	18,300
	Std (5)	1,800	300	70	100	600	310	1,400	300
	Condensate	280		320	90				
	Std (5)	50		40	10				
	Mass balance	98.5%		96%					
IF 2	MS	74,000	3,300	150	1,600	60,200	43,800	50,900	40,300
	Std (4)	3,600	900	20	800	8,700	6,700	7,100	7,100
	Fermentate	58,400	14,300	1,340	5,000	43,900	31,800	35,900	25,000
	Std (5)	900	700	200	800	3,400	2,500	2,600	2,500
	Condensate	1,210		250	420				
	Std (4)	230		20	20				
	Mass balance	83%		76%					

^a^ Std is the standard deviation of the parameter in the preceding row.

^b^ The value in the brackets is the number of samples used to determine the average and standard deviation.

^c^ The ratio of the measured parameter to the expected amount in the effluent.

**FIGURE 3:**
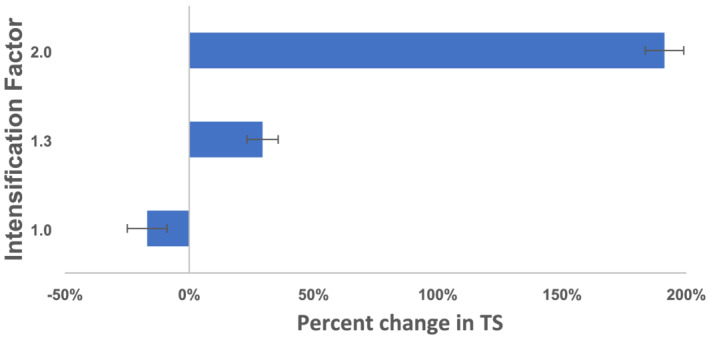
Percentage change in TS concentration of fermentate to mixed sludge at the termination of the experiment. Sample collected after final vacuum extraction for IF 1.3 and IF 2

The changes observed in the TS concentrations of MS fed into each system compared to the TS concentrations of the fermentate flow out of the system are shown in Figure 3. A reduction in TS concentration was noted in the control reactor, while thickening was achieved using the IC system. The IF 1.3 fermentate thickened by 30%, while the IF 2 fermentate was concentrated by 190%. The reduction of TS concentrations in a conventional fermentation is expected. Yuan et al. (2010) observed 21% destruction of solids in MS fermented using 4‐day SRT. Although the experiment was conducted at 22°C, the concentration of TS was 14 g/L, less than 50% of the present study.

**FIGURE 4:**
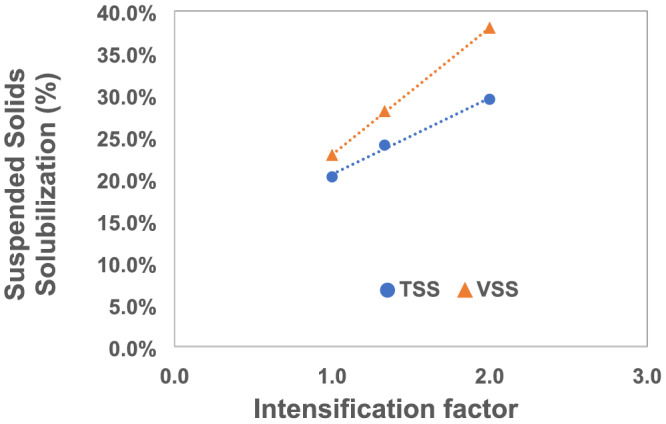
Percentage solubilization of total and volatile suspended solids observed in reactors at different intensification factors

Figure 3 confirms one of the central claims associated with the IntensiCarb technology: the possibility of combining fermentation and sludge thickening in a single vessel. It should be noted that the data reported in Figure 3 are the combined effect of evaporation due to the vacuum application cycle and long‐term thickening associated with fermentation. Moreover, the average daily solubilization of TSS and VSS after IC fermentation was determined and is shown in Figure 4. Remarkably, the solubilization of TSS and VSS observed increased linearly with IF, confirming that the process can not only be operated a reduced HRT but also with higher yields. The peak destruction of 29.5% and 38% for TSS and VSS, respectively, was observed in the IF 2 fermenter, that is, when the fermentation process is operated in half the volume of a conventional digester using the IntensiCarb technology. Conversely, the lowest destruction was observed in the control reactor (TSS, 20%; VSS, 23%).

VSS showed higher solubilization than TSS in all fermenters, with the difference increasing with intensification. The extent of solubilization of TSS and VSS is similar to those observed in other studies. For example, when MS was pretreated with thermal hydrolysis (170°C, 30 min) and fermented at thermophilic conditions with SRT of 3 days, Koupaie et al. (2021) found TSS and VSS solubilization of 33% to 34% and 41% to 43%, respectively. The solubilization of TSS is lower than VSS because of inert suspended solids that do not degrade in the fermenters. The accumulation of inert suspended solids could be because more non‐biodegradable material is fed with more significant intensification (Koupaie et al., 2021). It has been reported that the destruction of VSS increases with SRT. However, VSS solubilization observed in the IC units is comparable to VSS solubilization of 24% to 40% noted in the fermentation of WAS at 22°C and up to 7‐day SRT (Yuan et al., 2009). The use of IC resulted in similar solubilization performance to fermenters with longer SRTs and 65% better destruction than the conventional fermenter at the same SRT while operating at half the volume.

### Influence of intensification factor on sCOD, VFA, and ammonia yields

**FIGURE 5:**
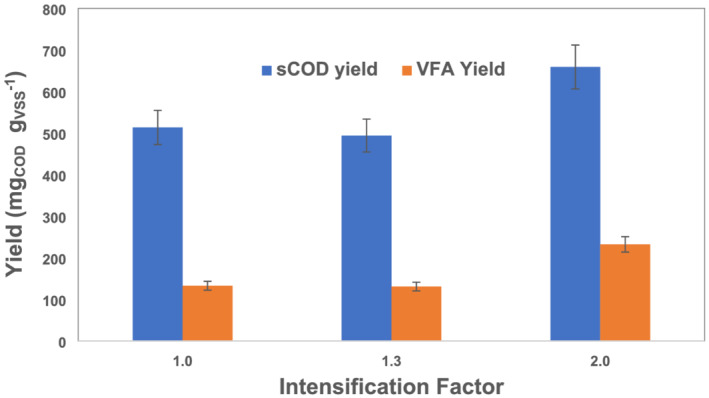
Average daily yield of soluble COD and total VFAs (expressed as COD) per mass of volatile suspended solids for different intensification factors

The effect of IC on sCOD yield and VFA yield of organic matter is shown in Figure 5. The yield of sCOD ranged from 500 to 660 mg COD/g VSS, while the VFA yield ranged from 133 to 233 mg VFA‐COD/g VSS. Both characteristics follow the same trend, with the maximum in the two cases being observed in the IF 2 fermenter. The IF 1 and IF 1.3 reactors did not show a significant difference in sCOD and VFA yield. During the experimental period, IF 1.3 and IF 2 condensates contained 145 to 285 and 145 to 479 mg COD/L VFA, respectively, comprising less than 7% of the VFA yield from both intensified reactors. The yield of sCOD observed in this study is comparable to another study performed using mixed sludge at thermophilic conditions (Koupaie et al., 2021). By applying various configurations of hydrothermal treatment and fermentation of 3‐day SRT, the researchers observed sCOD yield from 350 to 500 mg/g VSS. The similar values observed indicate that sCOD yield due to IC may be comparable to hydrothermal pretreatment. The VFA yield was comparable in their study, achieving up to 215 mg COD/g VSS.

**FIGURE 6:**
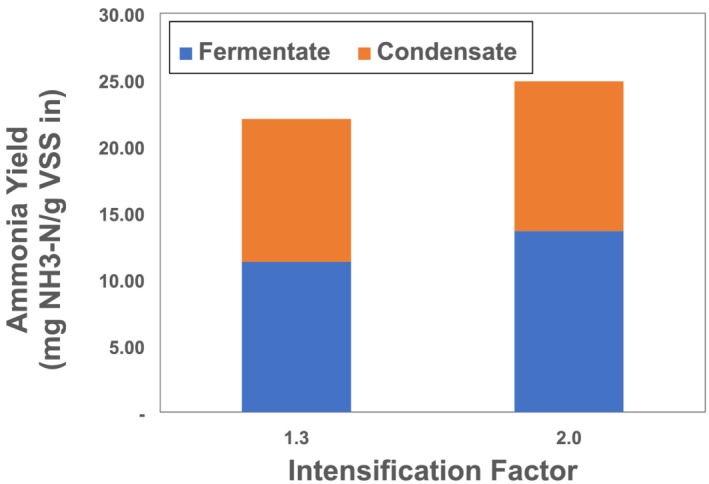
Fraction of ammonia yields in fermentate and condensate for intensified systems

Figure 6 shows the average daily ammonia yield for the two intensified fermentation experiments. As with the yield of soluble organic matter, the maximum ammonia yield of 25 NH_3_‐N mg/g VSS was observed in the IF 2 fermenter. A total of 49% and 46% of the total ammonia yield in the IF 1.3 and IF 2 tests, respectively, were extracted due to vacuum application. Florida DEP (2001) equations were used $f=\frac{1}{10^{\mathrm{pKa}-\mathrm{pH})}+1}$ and $\mathrm{pKa}=0.0901821+\frac{2729.92}{T_{k}}$ to determine the fraction of un‐ionized ammonia in the fermentate. In these equations, *f* is the fraction of unionized ammonia, *pKa* is the negative log of the acid equilibrium constant, *pH* is of the solution, and *Tk* is the temperature in degrees Kelvin. This un‐ionized fraction corresponds to the volatile fraction of ammonia. In the IF 1, IF 1.3, and IF 2 reactors, the un‐ionized ammonia fraction in the fermentate ranged from 5% to 8.4%, 6% to 22%, and 1.3% to 1.7%, respectively. The unionized ammonia fractions for the intensified fermenters were determined for fermentate after the vacuum had been applied. However, the ammonia mass stripped into the condensate was 6 and 14 times more than the unionized fraction of ammonia mass in IF 1.3 and IF 2 fermentate, respectively. The rate of ammonia extraction averaged throughout vacuum application was 180 and 80 mg N/h for IF 1.3 and IF 2 reactors, respectively. The high ammonia content in the condensate indicates that the vacuum may have driven the fraction in favor of un‐ionized ammonia production during application. Extraction of ammonia in the fermenter can maintain concentrations such that AD inhibition is prevented (Jeong et al., 2019). With pH adjustment, the ammonia extracted using IC could surpass 90%, corresponding to a significant nutrient resource as observed in a study in which ammonia was stripped from digestate and manure using vacuum thermal technology (Tao et al., 2018).

**FIGURE 7:**
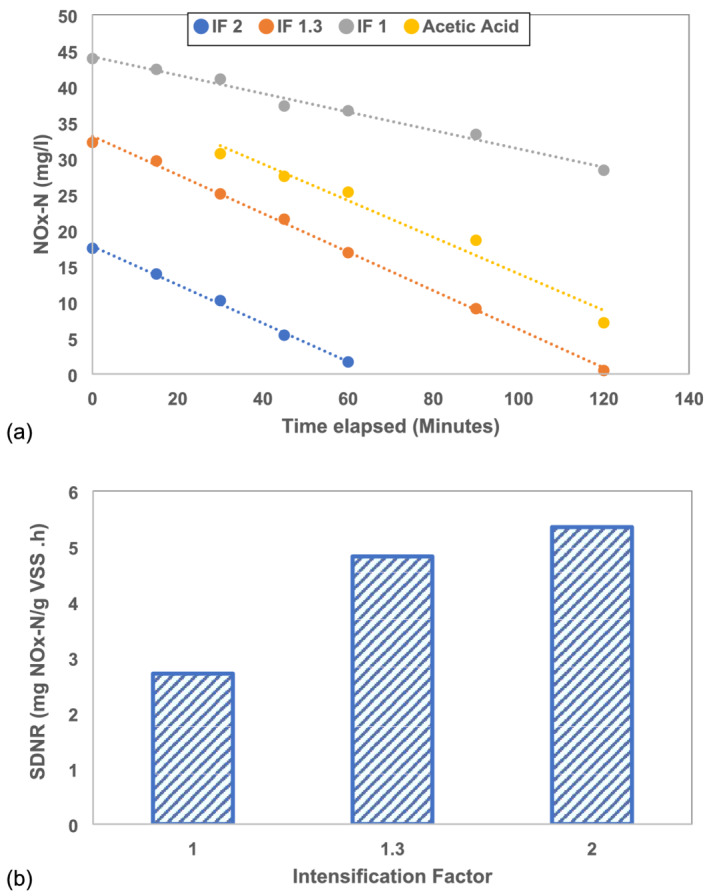
(a) Determination of maximum SDNR for all fermentates and acetic acid as carbon source; (b) maximum SDNR for fermentates of different intensification factors

The centrate of the fermentate and acetic acid was used as carbon sources for the SDNR test. The characteristics of these carbon sources and the resulting denitrification rate are presented in Table 3. The maximum SDNR for each reactor was determined as the slope of the linear trend of NOx depletion with time during the most rapid stage, as shown in Figure 7. Despite having less than 40% of the carbon available as VFA, the IF 1.3 and IF 2 fermentates yielded 5% to 17% higher denitrification rates than acetic acid. The similar observed rates are likely due to soluble substrates other than VFA contributing to the carbon utilized during denitrification. Soares et al. (2007) observed a 70% faster denitrification rate when fermented WAS was used as a carbon source than acetate. The IF 1 fermentate performed 40% worse than acetic acid as a carbon source.

**TABLE 3:** Properties of the mixture of WAS, carbon source, and deionized water in the reactor used for SDNR test

Parameters	Fermentate IF = 1	Fermentate IF = 1.3	Fermentate IF = 2	Acetic acid
Initial measured sCOD:N ratio	7.9	8.0	12.6	10.3
pH	7.75	7.60	6.85	7.06
NO_x_ $\left(\frac{\mathrm{mg}\;N}{l}\right)$ removed	35	28.6	17.1	31.2
SDNR $\left(\frac{\mathrm{mg}\;NO_{x}-N}{g\;\mathrm{VSS}.h}\right)$	2.71	4.82	5.35	4.57

The maximum denitrification rate observed in the conventional fermenter for this study appears consistent with other studies (Yuan et al., 2019), where pretreated, fermented, and filtered WAS was used as the carbon source achieving a nitrate utilization rate of 2 to 3 mg NO_3−_N/g VSS.h. Similarly, another study using the 2‐day fermented WAS in a sequencing batch reactor for denitrification achieved 2.4 to 3.2 mg NOx‐N/g VSS.h at HRTs from 6 to 8 h (Guo et al., 2017). However, some improvement in denitrification was observed due to intensification. It is hypothesized that the intensification could make a fraction of soluble organic matter more bioavailable for denitrification.

## CONCLUSION

This proof‐of‐concept study was performed to explore the potential of the IntensiCarb technology to intensify conventional anaerobic biosolids treatment technologies. The IntensiCarb unit was used to decouple the 3‐day SRT to HRT of 2.25 and 1.5 days in the fermentation of mixed sludge. The solids concentration in the mixed sludge was thickened more than two times during the intensified 1.5‐day HRT operation. The reduction of VSS was enhanced, and solubilization of organic matter was improved. The increased solubilization of organic matter allowed the fermentate to be used as a carbon source for denitrification. The fermentate from both IC units resulted in higher denitrification rates than even acetate. The IC system also proved capable of extracting ammonia into a high‐quality condensate stream used in nitrogen recovery. As the system accumulates particulate and non‐volatile dissolved sludge constituents, modeling studies will be needed to assess risks associated with the accumulation of these critical components. This study confirmed that nutrient recovery in the condensate is dictated by temperature and pH. Further studies can be performed to elucidate process control for better recovery efficiency. Upcoming research into the technology will be conducted to investigate the economic impact of intensifying anaerobic digestion using the IC system.

## AUTHOR CONTRIBUTIONS

**Frances Okoye:** Data curation; formal analysis; investigation; visualization. **Farokh Laqa Kakar:** Investigation; validation. **Elsayed Elbeshbishy:** Conceptualization; methodology. **Kati Bell:** Funding acquisition; resources. **Christopher Muller:** Conceptualization; methodology. **Jose Jimenez:** Funding acquisition; resources. **Ahmed Al‐Omari:** Conceptualization. **Domenico Santoro:** Conceptualization; methodology; supervision. **Eunkyung Jang:** Project administration; validation. **John Walton:** Conceptualization; supervision. **Gholamreza Bahreini:** Validation. **Masuduz Zaman:** Investigation. **George Nakhla:** Conceptualization; methodology. **Ferenc Hazi:** Validation. **Imre Takacs:** Validation. **Sudhir Murthy:** Conceptualization; methodology.

## Data Availability

The authors will make research data available upon request.
